# The influence of immune checkpoint blockade on the outcomes of allogeneic hematopoietic stem cell transplantation

**DOI:** 10.3389/fimmu.2024.1491330

**Published:** 2024-11-20

**Authors:** Yalei Hu, Yuxin Wang, Kaili Min, Huisheng Zhou, Xiaoning Gao

**Affiliations:** ^1^ Senior Department of Hematology, the Fifth Medical Center of PLA General Hospital, Beijing, China; ^2^ Graduate School, Chinese PLA General Hospital, Beijing, China; ^3^ State Key Laboratory of Experimental Hematology, the Fifth Medical Center of PLA General Hospital, Beijing, China

**Keywords:** immune checkpoint inhibitors, programmed cell death protein 1, programmed cell death 1 ligand 1, allogeneic hematopoietic cell transplant, graft-versus-host disease, immune-related adverse events

## Abstract

The principle of immune checkpoint blockade therapy is based on the activation of T cells. Immune checkpoint inhibitors (ICIs), such as anti-PD-1/PD-L1 and anti-CTLA-4 antibodies, have demonstrated effectiveness in treating solid tumors by reinvigorating the immune system to recognize and eliminate malignant cells. In recent years, ICIs have shown promise in certain patients with relapsed or refractory lymphoma and myeloid malignancies. Allogeneic hematopoietic stem cell transplant (allo-HCT) currently remains the only curative immunotherapy option for eligible patients with these hematologic malignancies. An increasing number of patients with indications for allo-HCT have received treatment with ICIs either before the procedure or as a therapy for relapse after allo-HCT. Nevertheless, initial reports suggest that patients exposed to immune checkpoint inhibitors either before or after allo-HCT are at an increased risk of developing severe graft-versus-host disease and other immune-related adverse events, likely due to the persistent effects of immune checkpoint blocking. Maximizing therapeutic benefits while minimizing side effects of the combination of checkpoint blockade immunotherapy and allo-HCT is an active area of research aimed at improving the prognosis of relapsed or refractory hematologic malignancies. However, there is still a lack of rational design strategies to optimize the combined use of these two different types of immunotherapies. In this review, we addressed the scientific rationale behind ICIs for treating lymphoma and myeloid malignancies. We also summarized the evidence supporting the use of ICIs as salvage therapy before and after allo-HCT. Additionally, we offered insights into current approaches for preventing and treating graft-versus-host disease and other immune-related adverse events during the procedure.

## Introduction

The immune system is regulated by a precise system of checks and balances that mediate protective immunity against invading pathogens while maintaining self-tolerance (1). The immune checkpoints consist of several stimulatory and inhibitory mechanisms that control the function of cells within the innate and adaptive immune system (2). Stimulatory checkpoint pathways promote activation and proliferation of T helper cells or effector CD8^+^ T cells. Conversely, inhibitory checkpoint pathways can regulate the extent of T cell activation and duration of immune responses, thereby preventing autoimmune reactions (3). Tumors can hijack inhibitory immune checkpoints to facilitate immune escape, making them attractive targets for cancer immunotherapy. For instance, many tumor cells inappropriately express programmed cell death 1 ligand 1 (PD-L1) protein, enabling them to evade attack from immune killer cells. Blocking the T cell checkpoint inhibitors programmed cell death protein 1 (PD-1) or signaling PD-L1 on cancer cells has demonstrated remarkably durable clinical responses in certain cancers, as it releases T cells from checkpoint inhibition to unleash antitumor activity (4–8). The primary therapeutic modalities for checkpoint blockade therapy involve using monoclonal antibodies engineered to block interactions between immune checkpoint receptors and their ligands. This prevents the off signal and allows immune cells to eliminate cancer cells.

Despite the promising clinical efficacy in multiple cancer types, currently approved immune checkpoint inhibitors (ICIs) only benefit a subset of patients with hematologic malignancies (9, 10). Even among those patients who initially responded, relapse may have occurred eventually due to acquired resistance, indicating that ICIs alone are unlikely to be curative for the majority of patients (11–13). Allogeneic hematopoietic stem cell transplant (allo-HCT) remains the only potentially curative therapeutic modality for most patients with prior checkpoint blockade therapy (14, 15). However, concerns have been raised about the safety of using ICIs either before or after allo-HCT due to potential increased rates and severity of subsequent toxicity, such as graft-versus-host disease (GVHD) and other immune-related adverse events (irAEs) (16–18). Many questions remain regarding the implications of immunomodulatory effects of checkpoint blockade, the optimal prophylactic and therapeutic strategy for GVHD, as well as the safety and efficacy of checkpoint blockade therapy in those patients experiencing relapse after allo-HCT. The lack of prospective data makes these treatment decisions challenging, but clinicians caring for patients with hematologic malignancies must address these important questions in their routine practice.

In this review, we discuss the mechanisms of action of ICIs in lymphoma and myeloid malignancies. We present a summary of the evidence that supports the use of these agents as salvage therapy before and after allo-HCT, with a specific focus on transplant outcomes and methods for preventing GVHD. Furthermore, based on available data and clinical experience, we also provide our recommendations to assist clinicians in guiding their practice until more definitive evidence is obtained.

## Role of immune checkpoint inhibition in hematologic malignancies control

**Figure 1:**
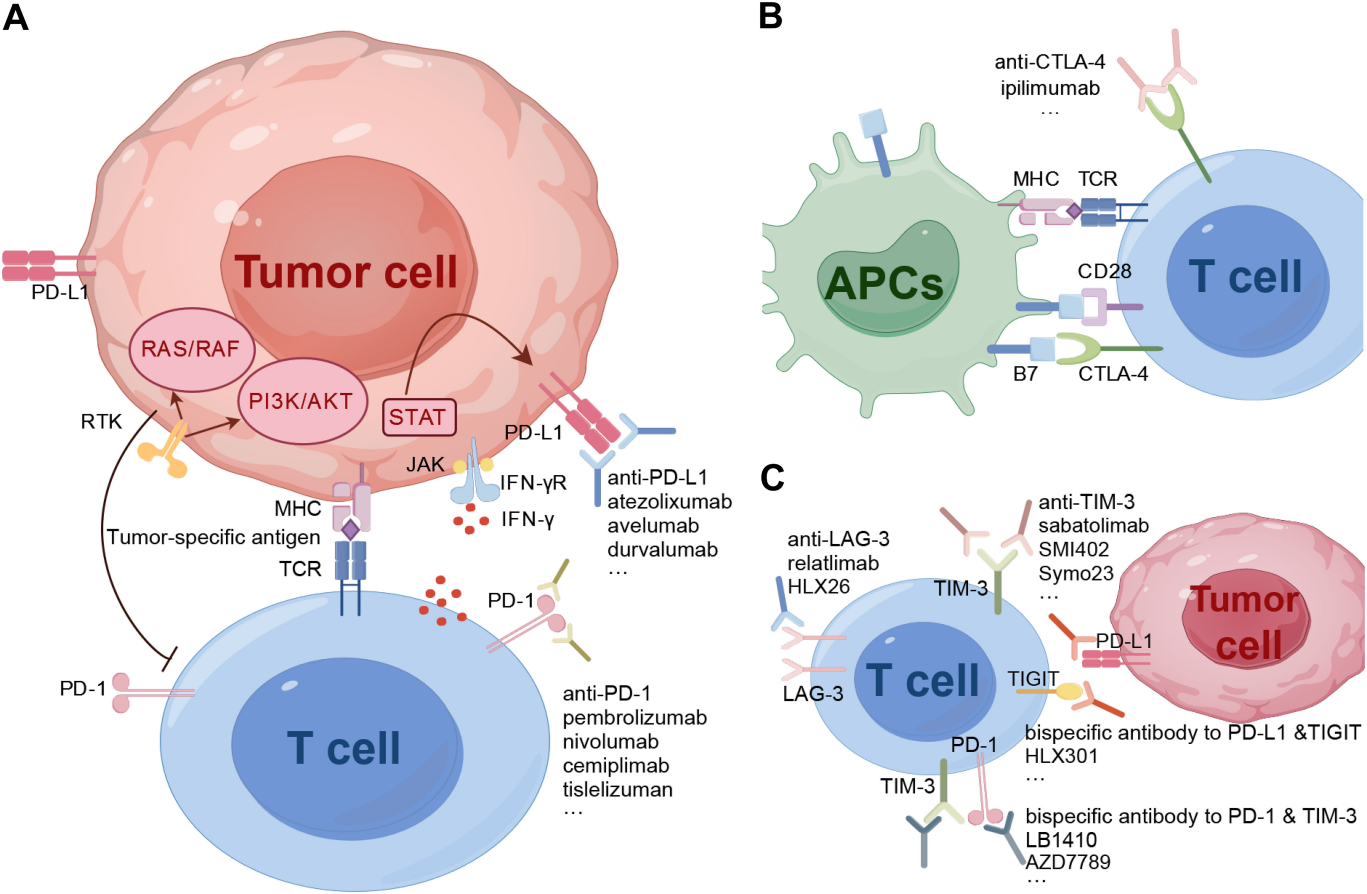
Inhibitory immune checkpoint molecules and emerging checkpoint inhibitors. **(A)**, IFNγ produced from T cells binds to the IFNγ receptor, leading to the regulation of PD-L1 expression through the JAK-STAT pathway. Both the JAK-STAT pathway and RTKs pathway have immunosuppressive functions. **(B)**, CTLA-4 competitively binds to B7, inhibiting the proliferation and activation of T cells. **(C)**, In addition to classical molecules, other immune checkpoints on T cells such as LAG-3, TIM-3, and TIGIT also contribute to immune suppression. Mechanisms of checkpoint inhibitors involve targeting these inhibitory checkpoint molecules expressed by T- and antigen-presenting cells. By blocking these receptors, immune checkpoint inhibitors promote the proper induction and differentiation of T cell-mediated immunity. APCs, antigen presenting cells; CTLA-4, cytotoxic T lymphocyte-associated protein 4; LAG-3, lymphocyte activation gene-3; MHC, major histocompatibility complex; PD-1, programmed cell death protein 1; PD-L1, programmed cell death protein ligand 1; RTK, receptor tyrosine kinase; TCR, T cell receptor; TIGIT, T cell immunoreceptor with immunoglobulin and ITIM domain; TIM-3, T cell immunoglobulin and mucin-domain containing-3.

During the past decade, immune checkpoint blockade therapies, including anti-PD-1 (pembrolizumab, nivolumab, cemiplimab and tislelizumab), anti-PD-L1 (atezolizumab, avelumab, and durvalumab), and anti-CTLA-4 (ipilimumab), have significantly revolutionized the treatment approach for hematologic malignancies. Furthermore, the ongoing discovery of novel immune checkpoints has led to the emergence of drugs targeting new molecules such as T-cell immunoglobulin and mucin domain 3 (TIM-3), Lymphocyte-activation gene 3 (LAG-3), and T cell immunoreceptor with immunoglobulin and ITIM domain (TIGIT) on a continual basis (**Figure 1**).

T cell activation is initiated by the recognition of peptides presented by major histocompatibility complex (MHC) molecules through their surface receptor, known as the T cell receptor (TCR). This recognition triggers the production of inflammatory cytokines and initiates the inflammatory response. The cytokines produced by activated T cells, such as interferon γ (IFN-γ) and tumor necrosis factor α (TNF-α), have the ability to up-regulate PD-L1 expression on tumor cells. The interaction between PD-L1 and PD-1 on immune cells leads to immune tolerance. Upon binding, the intracellular ITIM of PD-1 becomes phosphorylated and binds to the protein tyrosine phosphatase SHP-1, while the intracellular ITSM of PD-1 becomes phosphorylated and binds to the protein tyrosine phosphatase SHP-2. The recruited SHP-2 then dephosphorylates and inactivates downstream molecules in the TCR signaling pathway, including extracellular regulated protein kinase (ERK) and phosphatidylinositol-3-kinase (PI3K), thereby inhibiting TCR-mediated effector functions and cytotoxic T cell activity (19). The subsequent inhibition of TCR-mediated signaling activation and cell proliferation results in T-cell exhaustion. Zhou et al. have demonstrated that the coexpression of TIM-3 and PD-1 identifies a CD8^+^ T-cell exhaustion phenotype in mice with disseminated AML (20). Additionally, PD-1 has the ability to up-regulate genes that inhibit T-cell function, such as alkaline leucine zipper transcription factors ATF. This can ultimately lead to T cell depletion (21). Moreover, the activation of the PD-1/PD-L1 pathway can enhance the expression of IL-10 and result in dysfunction of T cell (22). Therefore, the inhibitory PD-1/PD-L1 checkpoint pathway plays an important role in the immune evasion of tumor cells. The strategies of anti-PD-1/PD-L1 immunotherapy aim to preserve the antitumor capacity of T cells by overcoming the PD-1/PD-L1 interaction, thereby inhibiting the immune escape of tumor cells. These strategies show promise in the treatment of R/R hematologic malignancies. In classic Hodgkin’s lymphoma (cHL), the common copy number gain of chromosome 9p24.1 leads to the upregulation of PD-L1 expression on Reed-Sternberg cells, prompting the systematic development of PD-1 checkpoint inhibitors in hematologic malignancies (23). Furthermore, the expression of PD-L1 was found to be up-regulated on murine leukemia C1498 cells when they were grown *in vivo*. When PD-1 knock-out mice were challenged with C1498 cells, they exhibited enhanced antitumor T-cell responses, reduced AML burden in the blood and tissues, and had significantly prolonged survival compared to wild-type mice. Similar outcomes were observed following treatment with anti-PD-L1 antibody therapy (24). Additionally, the frequency of PD-1^+^/CD8^+^ T cells was found to be higher in bone marrow samples obtained from patients with multiply relapsed AML compared to those with first relapsed or newly-diagnosed AML (25). Significantly, the high expression of PD-1, PD-L1, and PD-L2 in bone marrow samples from patients with AML has been linked to poor overall survival (OS) (26). These findings establish a scientific rationale for the utilization of PD-1 or PD-L1 antibodies in the treatment of AML patients.

CTLA-4 is a crucial immune checkpoint molecule predominantly expressed on activated T cells and regulatory T cells (Tregs). It plays a pivotal role in inhibiting T-cell activation and regulating immune homeostasis. In both *in vivo* and *in vitro* T cell responses, overexpression of CTLA-4 has been shown to inhibit T cell activation, which is dependent on the interaction between B7 and CD28. Furthermore, CTLA-4 on T cells has the ability to capture B7 molecules from antigen-presenting cells (APCs), leading to degradation of B7 ligands and subsequently inhibiting the CD28/B7 costimulatory pathway (27). CTLA-4 binds to B7 and triggers reverse signal transduction, leading to the activation of indoleamine 2,3-dioxygenase (IDO). This in turn induces tryptophan catabolism, thereby inhibiting T cell proliferation and activation (28, 29). Therefore, the CTLA-4/B7-mediated immune checkpoint in tumor microenvironment (TME) is another important component of the tumor immune escape. Therefore, the high expression of CTLA-4 in TME will inhibit proliferation and activation of T cells through multiple mechanisms, inducing tumor immune escape. Anti-CTLA-4 antibody can increase CD28 signaling in T cells, prevent Treg cell-mediated B7 endocytosis, and enable T cells to be activated. Saudemont et al. demonstrated that blocking B7/CTLA-4 interaction enhanced cytotoxic T cell-mediated killing of the leukemia cells in the DA1-3b murine AML model and prolonged the survival of mice (30). In cHL, there is a greater enrichment of CTLA-4 positive T cells, with their numbers exceeding that of PD-1^+^/LAG-3^+^ T cells (31).

Apart from CTLA-4 and PD-1, the upregulation of other immune checkpoint molecules such as LAG-3, TIM-3, and TIGIT has been confirmed (32). LAG-3, which shares structural similarities with CD4, competes for binding to MHC class II molecules, resulting in decreased efficiency of antigen presentation and thereby inhibiting the anti-tumor response. A study has demonstrated that T cell dysfunction could be reversed by removing the LAG-3 expressed Tregs in HL (33). TIM-3 expressed on myeloid leukemia cells interacts with ligands on T cells and suppresses anti-tumor immunity (34), which is correlated with poor clinical outcomes (35). Sabatolimab is an inhibitor of TIM-3 demonstrating effects against leukemia and is presently being studied in clinical trials (36).

## The use of immune checkpoint blockade prior to Allo-HCT

### Prior exposure to ICIs before allo-HCT is associated with better survival but also higher risk of GVHD

Immunotherapy has demonstrated efficacy in the treatment of malignant lymphomas, particularly in cHL and primary mediastinal B-cell lymphoma (PMBCL). A study involving 243 relapsed or refractory (R/R) cHL patients treated with nivolumab showed an overall response rate (ORR) of 71.2%, with a complete response (CR) rate of 21.4%. Notably, the median OS was not reached, and the 5-year OS was 71.4% (37). The phase 2 KEYNOTE-087 trial, which involved 210 participants with R/R cHL, reported an ORR of 71.9% and a CR rate of 27.6% with pembrolizumab treatment. The median OS was not reached, and the progression-free survival (PFS) was 13.7 months (38). Apart from PMBCL, the outcomes following ICIs in Non-Hodgkin Lymphoma (NHL) have been disappointing, with few durable responses. The phase 1b KEYNOTE-013 study reported an ORR of only 22% (19/86) in R/R NHL patients who received pembrolizumab. Furthermore, when considering disease type, the highest ORR was observed in PMBCL at 48% (10/21) (39). Despite the significant promise shown by PD-1 inhibition in enhancing ORR, a substantial proportion of patients do not experience lasting effects from this treatment. As a crucial therapeutic option for hematological malignancies, allo-HCT offers a viable choice for young patients who do not achieve CR after undergoing therapy with PD-1 blockade.

Allo-HCT following PD-1 blockade is linked to enhanced survival, but it also carries an increased risk of GVHD or other irAEs. Merryman RW et al. identified 31 patients with HL who underwent PD-1 blockade followed by allo-HCT. The 1-year OS and PFS were 90% and 74%, respectively. However, the cumulative incidence rates for grade 3-4 acute GVHD were found to be at 26%, with a rate of 13% for grade 4 acute GVHD, both figures markedly exceeding previous reports of 23% for grades 2-4 acute GVHD and 8% for grades 3-4 acute GVHD (40). Additionally, 3 patients developed severe hepatic veno-occlusive disease (VOD), and 7 patients experienced febrile syndrome (16). Another retrospective study reported on 13 patients with R/R cHL who underwent allo-HCT after PD-1 blockade therapy. At the time of allo-HCT, 4 patients achieved CR, 7 achieved partial remission (PR), and 2 had persistent disease. Interestingly, all patients achieved CR following allo-HCT. The OS and PFS at 57.4 months were 90.9% and 75.5%, respectively. However, acute GVHD developed in 5 patients (38.5%) within 3 to 5 weeks after transplantation, and non-infectious fever occurred in 7 patients (54%) (41).

Data regarding NHL patients undergoing allo-HCT following treatment with ICIs is scarce, as this information is primarily included in larger clinical studies focused on HL patients (16, 41). In investigations of ICIs for NHL, a limited number of patients proceed to allo-HCT; however, detailed data on these cases is not provided (42–45). In the KEYNOTE-013 and KEYNOTE-170 studies, 9 patients with R/R PMBCL underwent subsequent HCT following treatment with pembrolizumab. Among the 4 patients who received autologous HCT (auto-HCT), all were alive and in ongoing CR. Of the 5 patients who received allo-HCT, 3 remained alive with no evidence of disease, 1 survived but experienced disease recurrence, and 1 succumbed to disease progression at data cutoff (43). In another study, CheckMate 436, 12 patients with R/R PMBCL received allo-HCT (n=6) or auto-HCT (n=6) following treatment with nivolumab plus brentuximab vedotin (BV). At the 100-day after transplantation, 1 patient died due to neurological complications, while the remaining 11 patients were alive with CR. The 2-year CR rates were 80% for allo-HCT and 100% for auto-HCT. These results suggest that ICIs could be a viable strategy for bridging to auto-HCT or allo-HCT in patients with R/R PMBCL (45).

For many patients diagnosed with AML or myelodysplastic syndromes (MDS), allo-HCT remains the primary treatment strategy aimed at achieving a cure. However, there is limited data available on the effectiveness and safety of allo-HCT following checkpoint inhibition therapy in patients with AML or MDS (46). We have conducted a prospective study that included 27 patients with R/R AML. These patients received a combination of the PD-1 inhibitor tislelizumab, along with a hypomethylating agent (HMA) and the CAG (cytarabine, aclarubicin/idarubicin, granulocyte colony-stimulating factor) regimen. Among these patients, 11 proceeded to allo-HCT. In our study, 5 patients (45.5%) experienced acute GVHD (grade 1-2 acute GVHD n = 3, grade 3-4 acute GVHD n = 2), and 1 patient (9.1%) developed mild chronic GVHD. Unfortunately, 1 patient died from grade 4 gastrointestinal tract GVHD on day 36 after transplantation. A total of 5 patients remained alive and in remission at the follow-up time points ranging from 6.8 to 22 months post-transplantation (47). In a subsequent study, the safety and efficacy of allo-HSCT were assessed in 15 R/R AML patients (12 haploidentical [HIDs], 2 matched siblings, 1 unrelated donor) who received the tislelizumab + HMA + CAG regimen. Among the HIDs patients, four received GVHD prophylaxis with anti-thymocyte globulin and reduced-dose posttransplant cyclophosphamide (PTCy). With a median follow-up of 20.9 months, the 2-year OS and GVHD-free/relapse-free survival rates (GRFS) were found to be 54% and 48.6%, respectively. Notably, no deaths or relapses were observed in the PTCy group, suggesting a potential survival benefit associated with using PTCy for GVHD prophylaxis in HID-HCT. The regimen demonstrated tolerability along with promising efficacy for bridging to allo-HSCT in R/R AML (48).

### PTCy-based GVHD prophylaxis mitigates GVHD associated with exposure to ICIs prior to allo-HCT

Multiple studies have demonstrated the efficacy of PTCy in preventing GVHD in individuals undergoing ICIs treatment (**Table 1**). Schoch LK et al. (17) conducted a retrospective study of 14 patients who underwent reduced-intensity conditioning (RIC) and received PTCy as GVHD prophylaxis, including 10 patients with HL, 2 patients with AML/MDS, and 2 patients with NHL. Importantly, none of the patients experienced grade 3 to 4 acute GVHD or chronic GVHD. At the end of the study, all 10 HL patients remained free from disease progression, with a median follow-up of 12.7 months. Similarly, a retrospective study compared 34 patients with R/R HL, of whom 10 received nivolumab before allo-HCT and 24 did not. Both groups received PTCy for GVHD prophylaxis. Only 1 patient in each group experienced grade 3 acute GVHD. The incidence of acute GVHD and chronic GVHD showed no statistically significant difference between the two groups. Furthermore, the OS in the nivolumab group was superior to that of the control group (80% *vs* 41.7%) (49). This study suggests that the use of PTCy alleviate the increased risk of GVHD associated with ICIs.

**Table 1:** Studies showing improved outcomes with PTCy-based GVHD prophylaxis in Allo-HCT with prior ICI exposure.

Author	Diagnosis	Drug	Days* (range)	Donor type	Conditioning regimen	GVHD prophylaxis	GVHD	irAEs	Best response/Survival outcomes
Schoch LK et al. (17) 2018	HL (10) NHL (2) AML/MDS(2)	Nivo (8) Ipi (3) Pembro (2) Nivo + ipi (1)	42 (18-212)	MUD (2) MMUD (2) HID (10)	RIC (14)	PTCy (14)	Grade 2 GVHD: 42.8% Grade 3-4 GVHD: 0%	No irAEs	At the conclusion of the study, the 10 HL patients remain without disease progression.
Ito A et al. (53) 2020	HL (25)	Nivo (23) Pembro (2)	59 (23–539)	MRD (8) MUD (2) MMUD (8) HID (5) Cord blood (2)	RIC (16) MAC (9)	PTCy (8) ATG (7)	Grade 2-4 aGVHD: 47% Grade 3-4 aGVHD: 17% moderate-to-severe cGVHD: 34%	non-infectious fever (15)	1-year OS: 81.3% 1-year PFS: 63.7%
De Philippis C et al. (58) 2020	HL (59)	Nivo (28) Pembro (1) No-ICIs (30)	60 (27-372)	HID (59)	ICIs NMA (21) RIC (8) No-ICIs NMA (24) RIC (6)	PTCy (59)	ICIs *vs* no-ICIs Grade 2-4 aGVHD: 41% *vs* 33% (*p*=0.45) Grade 3-4 aGVHD 3.4% *vs* 3.3% moderate-severe cGVHD: 7% *vs* 8% (*p*=0.67)	ICIs: non-infectious fever (3) no-CPI: VOD(1)	ICIs *vs* no-ICIs 2-year OS: 77% *vs* 71% 2-year PFS: 78% *vs* 53%
Merryman RW et al. (50) 2021	HL (209)	Nivo (168) Pembro (41)	81 (17–1029)	MRD (49) MUD (57) MMUD (9) HID (91) Cord blood (2) Haplo + cord (1)	RIC (121) NMA (72) MAC (16)	PTCy (112) no PTCy (91) ATG + PTCy (6)	PTCy *vs* no-PTCy: Grade 2-4 aGVHD 33% *vs* 41% (*p*=0.22) Grade 3-4 aGVHD 14% *vs* 18% (*p*=0.34) cGVHD 25% *vs* 46% (*p*=0.002)	Hepatic VOD (6) non-infectious fever (59)	2-year OS/GRFS/PFS All patients: 82/47/69% haplo/PTCy: 85/60/80% non-haplo/PTCy: 96/59/74% non-haplo/no PTCy: 78/59/60%
Wang et al. (48) *2023*	AML (15)	Tislelizumab	75 (51-207)	MRD (2) MMUD (1) HID (12)	MAC	ATG ATG + a reduced-dose of PTCy	aGVHD: grade 2-4 40%; grade 3-4 13.3%; 2-year incidence of moderate-to-severe cGVHD:10%	Grade 3 irAEs:2 (thyroiditis and pneumonitis)	2-year OS: 54%; 2-year and GRFS: 48.6%; median follow-up: 20.9 months (range, 1.2-34.2)
İskender D et al. (49) 2022	HL (34)	Nivo (10) No-Nivo (24)	38 (19-85)	Nivo MRD (7) MUD (2) HID (1) no-Nivo MRD (18) MUD (5) HID (1)	RIC	ATG + PTCy (34)	Nivo *vs* no-Nivo aGVHD (*p*=0.11) 30% *vs* 8.3% Grade 3 aGVHD 20% *vs* 4.2% cGVHD 30% *vs* 29.2% (*p*=1)	NA	At the 30-month follow-up after allo-HCT, the OS and PFS were 80% and 60%, respectively.
Oran B et al. (52) 2020	AML/MDS (43)	Nivo (32) Ipi (9) both (2)	63 (7-386)	MRD (7) MUD (25) HID (8) Cord blood (3)	PTCy: MAC/RIC 11/10 no-PTCy: MAC/RIC 11/11	PTCy (22) no-PTCy(21)	PTCy *vs* no-PTCy Grade 3-4 aGVHD: 5% *vs* 22% (*p*=0.2)	Grade 3 irAEs(3) Grade 2 irAEs(2)	PTCy *vs* no-PTCy 1-year OS: 81% *vs* 33% 1-year PFS: 56% *vs* 25%
Tschernia NP et al. (51) 2021	AML (9)	Pembro (9)	66 (23-287)	MRD (3) MUD/MMUD (4) HID (2)	MAC (4) RIC (5)	PTCy (4) No-PTCy (5)	Grade 2 aGVHD: 66% mild cGVHD: 22% PTCy group (4) Grade 2 aGVHD: 25% cGVHD: 0%	NA	1-year OS: 67% 1-year PFS: 44%

* The median time between the final administration of CPI treatment and the commencement of allo-HCT.

aGVHD, Acute graft-versus-host disease; allo-HCT Allogeneic hematopoietic stem cell transplant; AML, Acute myeloid leukemia; cGVHD, Chronic graft-versus-host disease; CIR, Cumulative incidences of relapse; CR, Complete response; GRFS, GVHD-free and relapse-free survival; HID, Haploidentical donor; HL, Hodgkin lymphoma; ICIs, Immune checkpoint inhibitors; Ipi, Ipilimumab; irAEs, Immune-Related Adverse Events; MAC, Myeloablative conditioning; MDS, Myelodysplastic syndrome; MMUD, Mismatched-unrelated donor; MRD, Matchedrelated donor; MUD, Matched-unrelated donor; NHL, Non-Hodgkin lymphoma; Nivo, Nivolumab; NMA, Non-myeloablative conditioning; NRM, Nonrelapse mortality; ORR, Objective response rate; OS, Overall survival; Pembro, Pembrolizumab; PFS, Progression free survival; PTCy, Posttransplant cyclophosphamide; RIC, Reduced intensity conditioning; VOD, Veno-occlusive disease. NA, Not Applicable

To determine whether the application of PTCy could reduce the incidence of GVHD in patients with HL, Merryman RW et al. conducted a study involving 209 HL patients who had undergone allo-HCT following prior treatment with PD-1 blockade. Of these patients, 112 (54%) received PTCy-based GVHD prophylaxis. Six patients developed grade 2-4 acute GVHD within the first 14 days following allo-HCT, and none of them had received PTCy prophylaxis. The incidence of grade 2-4 and grade 3-4 acute GVHD was 33% and 14%, respectively, in the patients who received PTCy. Multivariable analyses revealed that the use of PTCy for GVHD prophylaxis was associated with a significantly lower risk of chronic GVHD (25% *vs* 46%), but not with a reduced risk of acute GVHD (33% *vs* 41%). The only significant factor predicting the occurrence of acute GVHD was the time from the last dose of anti-PD-1 antibody to transplantation. Importantly, PTCy-based GVHD prophylaxis was associated with improved PFS and GRFS (50).

Similarly, PTCy-based GVHD prophylaxis mitigates severe GVHD in AML/MDS patients. In a retrospective analysis, 9 patients with R/R AML treated with pembrolizumab followed by allo-HCT, including 4 patients with PTCy-based GVHD prophylaxis. The 100-day mortality was 0%. Six patients (67%) developed grade 2 acute GVHD and no patients had grade 3-4 acute GVHD. The 1-year OS and PFS after allo-HCT were 67% and 44%, respectively. The median OS was 21 months with a median follow-up of 23 months (51). Oran B et al. conducted a study on the treatment outcomes of allo-HCT in 43 AML/MDS patients who received nivolumab (n=32), ipilimumab (n=9), or both (n=2) as part of their treatment regimen. The patients were divided into two groups based on the application of PTCy. In the PTCy group, consisting of 22 patients, the 100-day cumulative incidence of grade 3-4 acute GVHD was 5%, compared to 22% in the no-PTCy group. The results showed that in the PTCy group, the1-year OS and PFS rates were 81% and 56%, respectively. In contrast, the no-PTCy group had markedly inferior outcomes, with 1-year OS and PFS rates at 33% and 25%, respectively. Furthermore, only 5 patients experienced irAEs, including 3 grade 3 irAEs and 2 grade 2 irAEs (52). These findings suggest that the utilization of PTCy may improve the outcomes of transplantation without elevating the incidence of GVHD in AML/MDS patients who had received ICIs treatment prior to allo-HCT.

The above evidence (16, 17, 50, 53–56) demonstrates that allo-HCT following treatment with anti-PD-1 antibodies is associated with overall favorable outcomes. The majority of studies support the use of PTCy-based GVHD prophylaxis, which has been linked to prolonged survival and a reduced likelihood of developing GVHD (17, 50, 57, 58). Unlike PTCy, *ex vivo* T cell depletion (TCD) of the allograft represents an alternative strategy for the prevention of GVHD, obviating the need for post-transplant immunosuppressive therapy. There are limited reports regarding the incidence of GVHD in the TCD platform. Casadei B et al. (41) conducted a retrospective case series involving 13 patients with R/R cHL who were treated with an anti-PD-1 monoclonal antibody prior to allo-HCT. All but one patient received a T-cell-depleted graft, and ATG was included in the conditioning regimen for eight patients. The overall incidences of acute and chronic GVHD were found to be 38.5% and 23.1%, respectively. Notably, five out of the thirteen patients experienced acute GVHD, with only one patient presenting with grade II-III severity. A case report by Kim YE et al. (59) detailed the successful treatment of a patient with NK/T-cell lymphoma using pembrolizumab, followed by ex vivo TCRαβ-depleted haploidentical allo-HCT, resulting in sustained remission. The patient did not receive pharmacologic GVHD prophylaxis and did not experience acute GVHD. Given the limited sample size, we are currently unable to determine which of the two GVHD prophylaxis platforms has a lower incidence of GVHD at present, and further investigation and confirmation are required in prospective controlled trials. Additionally, since PTCy may be used in combination with ATG or followed by other immunosuppressive agents, we also cannot determine whether the reduction in GVHD incidence following the use of PTCy-based GVHD prophylaxis is related to other drugs. Larger prospective studies will be necessary to determine the effectiveness of PTCy in combination with other drugs in preventing GVHD, as well as the optimal interval between the last dose of PD-1 inhibitor administration and allo-HCT in order to minimize the occurrence of severe immune-related complications (58).

### The mechanism by which PTCy alleviates GVHD

GVHD occurs when the immune cells from a donor recognize the recipient’s allogeneic peptide-HLA as foreign, and the risk of GVHD increases with greater HLA disparity between the donor and recipient. This can lead to an alloreactive response that induces GVHD (60). PD-1, functioning as a coinhibitory receptor, modulates the proliferation, differentiation, and survival of T-cells upon interaction with its ligand, PD-L1. Ikegawa et al. (61) demonstrated that PD-1 blockade promoted sustained proliferation of alloreactive T cells while also promoting a temporary increase in Tregs proliferation. However, within two weeks, the number of Tregs decreases due to elevated susceptibility to apoptosis. This dysregulation of T cell subsets leads to severe GVHD. The authors also observed that alloreactive T cells from PD-1-knockout donor grafts were aggressively proliferative on day 3 and vulnerable to the cytotoxic effects of Cy. However, Tregs were resistant to PTCy because of aldehyde dehydrogenase expression (62). In conclusion, PTCy-based GVHD prophylaxis alleviates GVHD by rebalancing the T cell subsets (57, 61).

In conclusion, the decision to offer allo-HCT as a consolidation treatment option is based on factors such as age, disease status, prior therapies, comorbidity index score (HCT-CI), and donor availability. PD-1 therapy should be withheld for at least 6 to 8 weeks before allo-HCT. Discontinuing PD-1 therapy before transplantation requires careful assessment of any immune-related complications of the patient during treatment, monitoring for signs of disease progression or recurrence, a well-timed cessation plan, and a refined transplant strategy. The transplant strategy, including conditioning intensity and GVHD prophylaxis, should be optimized to reduce posttransplant immune toxicity. We recommend using RIC regimens for patients aged 60 years or older, those with heavy pretreatment, or HCT-CI scores of ≥3 due to the increased risk of VOD and GVHD with ablative conditioning regimens. PTCy-containing GVHD prophylactic regimen is preferred for patients with previous exposure to ICIs. Close monitoring is necessary for these patients as they are more likely to develop severe GVHD and noninfectious febrile syndrome. In cases of noninfectious febrile syndrome, IV methylprednisolone at 1 mg/kg per day should be considered. Rapid initiation of IV methylprednisolone at 2 mg/kg per day is recommended if GVHD occurs. For cases with previous exposure to ICIs and lack of rapid response to systemic steroids, an earlier transition from steroids to second-line therapies for GVHD should be considered compared to a context without checkpoint blockade.

## ICIs: an optional approach for relapse post allo-HCT

Recurrence remains the primary cause of transplant failure, primarily due to the loss of the graft-versus-tumor (GVT) effect, resulting in compromised outcomes (63, 64). The primary mechanism underlying immune escape involves the inhibition of allogeneic T cells, often through interaction with inhibitory receptors like PD-1 and CTLA-4 on their cell surfaces (63). Conventional treatments for relapse after allo-HCT, such as discontinuation of immunosuppressive drugs, donor lymphocyte infusion (DLI), salvage chemotherapy, and second transplantation, provide limited potential for achieving a cure and pose an increased risk of GVHD toxicity (65).

Previous reports suggest that ICIs after allo-HCT may reverse T-cell exhaustion and immune evasion, potentially enhancing the GVT effect (66). ICIs have shown significant clinical efficacy in the past decade, making them an attractive option for enhancing the GVT effect in managing post-transplant relapse after allo-HCT, especially in patients with classic cHL (67, 68). If used safely, ICIs could enhance GVT effects and reduce relapse after allo-HCT in lymphoma and potentially other hematological malignancies. Therefore, close monitoring is necessary for both irAEs and GVHD when using ICIs after allo-HCT (65). While most studies have focused on assessing ICIs after allo-HCT for HL, there is an increasing number of investigations into this strategy in other hematologic malignancies (**Table 2**). The majority of studies primarily focus on using one or two ICIs or their combination with demethylating agents for post-transplant relapse. There are few studies that explore the use of ICIs in combination with targeted drugs or DLI (69, 70).

**Table 2:** Immune Checkpoint Inhibitors for Relapse After Allogeneic HCT.

Author	Year	Study design	Diagnosis	Drug/Dose	Time after allo-HCT, median	Best response/ Survival outcomes	GVHD/Organ	GVHD Treatment	irAEs/Organ
Schoch L et al. (77)	2016	Retrospective	HL (4) AML (1) MDS (1) Solid cancer (3)	Nivo (6) Pembro (1) Ipi (3)	1.2 years	NA	No GVHD observed	NA	None
Herbaux C et al. (76)	2017	Retrospective	HL (20)	Nivo 3 mg/kg q2 weeks, median number of doses 8 (range,1-36)	23 months	12-month PFS: 58.2% 12-month OS: 78.7%	GVHD in 6/20 (30%), fatal in 2 (10%) patients	Corticosteroids +/- other therapies such as cyclosporine, basiliximab and photopheres	Hematologic AEs: grade 4 neutropenia and grade 3 thrombocytopenia (1); Nonhematologic AEs: grade 2 cerebellar ataxia (1)
Haverkos BM et al. (75)	2017	Retrospective	HL (29) NHL (2)	Nivo 3 mg/kg q2 weeks (n=28), pembro 200 mg q 3 weeks (n=3)	920 days	ORR 77% (15 CRs, 8 PRs), Median OS not reached	GVHD in 17/31 (55%), 6 acute GVHD, 4 overlap, 7 chronic GVHD; fatal in 8 patients	14 of 17 patients ≥2 systemic therapies	None
Manson G et al. (79)	2022	Retrospective	R/R HL (19) (Prior allo-HCT: 7; Prior auto-HCT: 11)	Nivo 3 mg/kg q2 weeks, median of 20 cycles of nivolumab	5.7 years	median PFS: 41.9 months; median OS: not reached; 5-year estimated PFS: 42.3%	No GVHD observed	NA	None
Holderried T et al. (70)	2019	Retrospective	MDS/AML (12) NHL (5) ALL (2) PMF (2)	Ipi (10) Nivo (5) Nivo + DLI (5) Nivo + Ipi (1)	59 days	Overall ORR/CR/PR: 43/14/29%; ORR: Ipi/Nivo/Nivo + DLI 20/40/80%; Overall median OS: 79 days; median OS: Ipi *vs* Nivo ( ± DLI) 39 days *vs* 282 day	Grade 3/4 acute GVHD or moderate/severe chronic GVHD developed in 6/21 (29%).	5/6 (83%) of Grade 3/4 acute GVHD or moderate/severe chronic GVHD were steroid refractory	None
Ito A et al. (53)	2020	Retrospective	HL (25)	Nivo (n=19, at 3 mg/kg or 240 mg/body in 13, 1.7 mg/kg in 1, and 0.5 mg/kg in 5), pembro (n=1, at 200 mg/body)	589 days	ORR: 75% CR: 40% 1-year OS: 89.7%	2-4 acute GVHD: 15%; moderate-to-severe chronic GVHD: 30%	About half of all patients had steroid-requiring ES or febrile syndrome	irAEs: 4/25 (16%), with grade 2 hypothyroidism in 3 (12%) and grade 3 interstitial pneumonia (IP) in 1 (4%)
Bashey A et al. (80)	2009	Phase Ib	HL 14 MM (10) AML (2) CLL (2) Other (5)	Single dose of ipi (ranging from 0.1 to 3 mg/kg)	366 days	ORR 10% CR 7% (HL)	No GVHD observed	NA	Organ-specific irAEs were seen in 4 patients
Davids MS et al. (25)	2016	Phase I/Ib	AML (12) HL (7) NHL (4) Others (5)	Ipi (ranging from 3 to 10 mg/kg every three weeks)	675 days	ORR 32% CR 23% (AML)	Total GVHD: 4 (14%); chronic GVHD of liver 3/28 (11%); acute GVHD of gut 1/28 (4%)	——	IrAEs were observed in 6/28 (21%), including one death (4%)
Khouri IF et al. (81)	2018	Phase II	MCL (3) CLL (2) FL (2) DLBCL (1) THL (1) ALCL (1)	Lenalidomide (10 mg/day for 21 days) + Ipi (3mg/kg, single dose) Repeated for 2 cycles	29 months	ORR/CR/PR: 70/40/30%; 90% OS at median follow-up of 20.5 months	No GVHD observed	NA	irAEs; asymptomatic hypothyroidism 1/10 (10%)
Godfrey J et al. (86)	2023	Phase I	AML (8) MDS (1) HL (1) DLBCL (2)	Pembro(200 mg every 3 weeks) for up to 2 years	587 days	ORR: 22%	No GVHD observed	NA	irAEs (any grade): 42%; grade 3 - 4 irAEs: 25% (pneumonitis, 2; hyperthyroidism, 1)
Davids MS et al. (90)	2020	Phase I/Ib	AML (10) MDS (7) HL (5) NHL (3) CLL (1) CMML (1) Leukemia NOS (1)	Nivo (q2w) 1 mg/kg: n = 6 0.5 mg/kg: n = 22	21 months	1 mg/kg: (ORR/CR/PR: 50/17/33%); 0.5 mg/kg: (ORR/CR/PR: 23/0/23%); median PFS/OS: 3.7/21.4 months	Chronic or acute GVHD occurred in 11/28 (39%) and was fatal in 2/28 (7%)	NA	Two of 6 patients treated at 1 mg/kg experienced DLT from irAEs; Twenty-two patients were treated at 0.5 mg/kg, and 4 DLTs occurred, including 2 irAEs and 2 with fatal GVHD.
Apostolova P et al. (92)	2023	Phase II	AML (16)	Nivo 3 mg/kg q2 weeks, median number of doses: 2 (range 1-7)	18.8 months	ORR (days 42): 25%; SD (days 42): 25%; median OS: 15.6 months.	Five patients (31.25%) developed grade I-II aGVHD, and four (25%) had cGVHD, including one fatal case.	NA	Nivolumab-related AEs grade 3 or higher: 4 patients (25%); In one case each, pericardial effusion, pleural effusion, pneumonitis, arthralgia, myalgia and encephalopathy were reported.
Garcia JS et al. (87)	2023	phase I	AML (23) MDS (2)	Ipi + Decitabine	——	ORR: 20%; median DOR: 4.46 months	Overall GVHD: 36%; Severe GVHD:8%	Steroid-responsive chronic GVHD developed at each tested IPI dose level in the post-allo-HCT arm, including 1 severe and 3 moderate cases.	Organ-specific irAEs were seen in 4/25 (16%) (Colitis, n=3; Pneumonitis, n=3)

ALCL, Anaplastic large T-cell lymphoma; allo-HCT, Allogeneic hematopoietic cell transplant; AML, Acute myeloid leukemia; CMML, Chronic myelomonocytic leukemia; CR, Complete remission; DLBCL, Diffuse large B-cell lymphoma; Nivo, Nivolumab; Ipi, Ipilimumab; Pembro, Pembrolizumab; DLI, Donor lymphocyte infusion; DLT, Dose-limiting toxicities; DOR, Duration of response; FL, Follicular lymphoma; GVHD, Graft-versus-host disease; HL, Hodgkin lymphoma; MCL, Mantle cell lymphoma; MDS, Myelodysplastic syndromes; NHL, Non-Hodgkin lymphoma; ORR, Objective response rate; OS, Overall survival; PFS, Progression-Free Survival; PMF, Primary myelofibrosis; PR, Partial remission; THL, Triple-hit lymphoma.

NA, Not Applicable

### Lymphomas

Early reports have indicated a high response rate with ICIs in HL patients relapsing after allo-HCT. However, it is important to note that an increased risk of sever GVHD and irAEs has been observed (69, 71–74). A retrospective study of 31 HL patients who underwent allo-HCT followed by treatment with nivolumab or pembrolizumab for relapsed disease showed an ORR of 77% and a CR rate of 50%. Notably, GVHD developed in 55% of the patients after receiving a median of 1 to 2 doses of treatment, including 5 patients without prior GVHD. Importantly, the majority of these patients showed poor response to systemic steroids, leading to complications related to GVHD and ultimately resulting in death for 26% or the cohort (75). Similarly, a multicenter retrospective analysis evaluated the efficacy of nivolumab at a dosage of 3 mg/kg every 2 weeks in HL patients who had relapsed after allo-HCT. Among the 20 identified patients, the ORR was 95%, and the median PFS had not been reached at a median follow-up of 370 days. Six out of the 20 patients (30%) experienced GVHD within one week following their initial dose of nivolumab, all with a history of acute GVHD, suggesting an increased level of allo-reactivity. Of these 6 patients, 3 were unresponsive to steroid treatment, and 2 ultimately succumbed to GVHD. Notably, the interval between allo-HCT and the first administration of nivolumab was shorter for those patients who developed GVHD compared to those who did not; with median times being 8 months versus 28 months, respectively (76). In contrast, another study found no instances of GVHD in 9 patients who received various ICIs after allo-HCT. It is noteworthy that the median time interval between allo-HCT and ICIs was 1.2 years, and all patients had received PTCy-based GVHD prophylaxis (77). Thus, it appears that a longer interval between the first administration of nivolumab and allo-HCT as well as PTCy-based GVHD prophylaxis are associated with reduced occurrence of GVHD.

While the potential association between GVHD and ICIs remains a significant concern, there have been documented cases demonstrating that effective management of GVHD can result in sustained remissions. A case report detailed the experience of a patient with refractory cHL who underwent two allo-HCTs (first from a matched unrelated donor, second from a haploidentical donor) following relapse on BV and nivolumab. Subsequently, BV-pembrolizumab combination therapy was administered for a relapse one year after the second transplant. Although moderate overlap GvHD emerged, necessitating treatment interruption, it was promptly brought under control. Notably, the patient maintained remission for over two years after just two cycles of BV-pembrolizumab combination therapy (78). A long-term study from the Early French Access Program revealed a nearly 40% CR rate in heavily pre-treated R/R HL patients, with 19 patients (25%) achieving remission without further consolidation after a median of 20 Nivolumab cycles. The median PFS was 41.9 months and the median OS was not reached. The 5­year estimated PFS was 42.3%. Importantly, 36.8% of patients (7/19) received anti-PD-1 therapy post-allo-HCT, demonstrating its feasibility in this challenging salvage setting (79).

Combination therapies, including ipilimumab in conjunction with other agents, have demonstrated promise in the treatment of post-allo-HCT lymphoma relapses, extending beyond the use of single-agent ICIs. A phase I trial with 29 patients found that a single dose of ipilimumab (0.1 to 3 mg/kg) showed positive responses in lymphoma patients, with no signs of GVHD or severe irAEs (80). In the following phase I/Ib study, higher doses of ipilimumab (3 to 10 mg/kg every three weeks) were given. Only the group receiving 10 mg/kg showed a response rate of 32%. There were 4 cases of GVHD and 6 irAEs, including 1 fatal case involving hepatitis and pneumonitis (25). Notably, patients with a history of grade 3 or 4 acute GVHD or autoimmune disease were excluded from these studies. Khouri et al. found that the combination of ipilimumab and lenalidomide showed promising results in 10 lymphoma patients who relapsed after allo-HCT, with 4 cases of CR and 3 cases of partial remission (PR), and no instances of GVHD (81).

### Myeloid malignancies

Treating relapsed AML after HCT remains challenging, as less than 20% of patients achieving lasting remission following chemotherapy combined with a second HCT or donor lymphocyte infusions (82). AML blasts from post-HCT relapse patients exhibit higher expression of inhibitory receptors (IRs) (83). In AML patients experiencing relapse after prolonged post-HSCT remission, functionally impaired CD8^+^ T cells expressing IRs such as PD-1, CTLA-4, and TIM-3 accumulate in the tumor microenvironment (84). This suggests the potential for ICIs to restore T cell activity and enhance anti-myeloid malignancy immune responses (85). Therefore, the utilization of ICIs for the management of recurrent myeloid malignancies following allo-HCT is increasingly recognized as a promising therapeutic strategy. Holderried et al. conducted a retrospective analysis of checkpoint blockade therapy for disease recurrence after allo-HCT other than HL, including 21 patients (MDS/AML n = 12, NHL n = 5, ALL n = 2, myelofibrosis n=2). The median follow-up was 59 days and the ORR was 43% (70). Patients who received DLI in combination with ICIs had a higher ORR of 80%. However, it is important to note that severe grade 3-4 acute GVHD and moderate to severe chronic GVHD were observed in 29% of all patients, with 83% being refractory to steroid treatment. Therefore, while the combination therapy of checkpoint blockade and DLI may be effective for relapsed patients after allo-HCT, it also poses a higher risk of severe GVHD. A recent Phase I study presented early results of pembrolizumab for treating relapse following allo-HCT (86). Among 9 patients with myeloid malignancies (8 AML, 1 MDS), pembrolizumab had limited effect, with only 2 patients showing stable disease (SD) (4 disease progression, 3 dis-continuation of pembrolizumab due to dose-limiting toxicity). These three participants (25%) with discontinuation of pembrolizumab experienced grade 3 to 4 irAEs, including pneumonitis in 2 patients and hyperthyroidism in 1 patient. All irAEs occurred after 1 to 2 cycles and resolved after discontinuation of pembrolizumab and corticosteroid treatment.

Ipilimumab-mediated CTLA-4 inhibition shows responses in about one-third of patients experiencing relapsed AML or other hematologic malignancies following allo-HCT, while maintaining a manageable safety profile (25, 87). In the phase I/Ib study, ipilimumab was given to 28 patients with relapsed hematological malignancies after allo-SCT, including 12 with AML. Among 22 patients with a dose of 10 mg per kilogram, 5 patients (23%) achieved a CR, including 3 with leukemia cutis, 1 with myeloid sarcoma, and 1 with AML secondary to MDS (25). A multicenter phase 1 trial (NCT02890329; CTEP 10026) is currently underway to investigate the combination of ICIs with decitabine treatment in patients with R/R MDS/AML, both in post-allo-HCT and transplant-naïve settings. Preliminary findings from this early study investigating the use of ICIs following allo-HCT for R/R AML suggest an ORR of 20% and an overall irAE rate of 44% (87). Transcriptomic analyses from the ETCTN/CTEP 10026 study, investigating the effects of decitabine and ipilimumab in AML/MDS patients post-HCT or in HCT-naive settings, have revealed a strong correlation between high baseline T cell-to-AML ratio and treatment response. Conversely, resistance is associated with inadequate clearance of diseased progenitor cells in post-HCT samples (88). Ipilimumab may facilitate regression of post-HSCT relapsed AML by recruiting cytotoxic CD8^+^ T cells to leukemic sites (88, 89). Further research is warranted to develop effective IPI-based treatment strategies that can achieve enduring responses while minimizing immune toxicity.

In AML patients with extramedullary disease relapse post-transplantation, nivolumab showed potential benefits in a multicenter prospective study. Nivolumab was administered every 2 weeks after allo-HCT for relapsed myeloid (n = 19) or lymphoid (n = 9) hematological malignancies. The initial dose of 1 mg/kg was de-escalated to 0.5 mg/kg due to observed toxicities. Among the patients treated at 1 mg/kg, 2 out of 6 experienced dose-limiting toxicity, while at the lower dose of 0.5 mg/kg, there were 4 cases of dose-limiting toxicity, including irAEs and fatal GVHD. The ORR in evaluable patients was 32% (8/25), with a one-year PFS and OS of 23% and 56%, respectively (90). A pilot study found unexpected severe toxicities in 4 patients with AML/MDS who received low-dose nivolumab as maintenance therapy post-allo-HCT. All 4 patients developed irAEs, with 2 experiencing serious adverse events, including grade 4 neutropenia and grade 3 autoimmune encephalopathy, leading to the termination of the study. It is noteworthy that the median time to drug administration from transplantation was 7.8 months, which may have contributed to increased toxicities (91). Even at low doses, nivolumab maintenance for myeloid malignancies in the post-allo-SCT setting may lead to serious irAEs beyond GVHD. Further research is needed to determine the appropriate dosage and timing of ICIs following allo-HCT (**Table 2**) (91). In a phase II, single-arm study, sixteen AML patients experiencing relapse following allo-HCT were administered HMA and nivolumab. The median number of nivolumab doses received was 2. At day 42, the ORR (CR/PR) was 25%, with an additional 25% achieving SD. The median OS was 15.6 months. Responders showed a higher frequency of activated (ICOS^+^, HLA-DR^+^), low-senescence (KLRG1^−^, CD57^−^) CD8^+^ effector T cells. AEs were consistent with previous studies, with GVHD being a common side effect. Five patients (31.25%) developed grade I-II aGVHD, and four (25%) had cGVHD, including one fatal case. This study suggests HMA/nivolumab treatment post-allo-HCT may dlicit anti-AML immune responses and could potentially serve as a bridge to a second allo-HCT (92).

A retrospective study was conducted on 21 patients with AML (n = 16) or MDS (n = 5) who received anti-PD-1 (16 patients) or anti-CTLA-4 (5 patients) therapy for disease relapse after allo-HCT. Despite early ICI therapy initiation, patients treated with PTCy had a lower observed cumulative incidence of grades 2 to 4 acute GVHD compared to those who did not receive PTCy (16% *vs*. 22%; *p* = 0.7). This suggests that PTCy may reduce the incidence of acute GVHD in ICI therapy for relapsed AML/MDS patients after allo-HCT (93). In theory, PTCy is thought to selectively eliminate primarily alloreactive activated T cells while preserving non-cross-reactive memory and naive T cells. It also promotes T regulatory cells and long-term tolerance through intrathymic clonal deletion of anti-host T cells (94–96).

In previous studies, ICIs were mainly used to treat hematologic relapse after allo-HCT. A recent study reported the first case of preemptive treatment using tislelizumab and azacitidine in a patient with MRD-positive AML. The patient received 100 mg of tislelizumab on day 1 and 100 mg of azacitidine on days 1-7 post-transplantation. After the combination therapy, RUNX1-RUNX1T1 transcripts became negative and complete donor chimerism was observed in bone marrow. However, the patient experienced moderate GVHD and irAEs affecting multiple organs, which were managed with immunosuppressive therapies. Using ICIs in MRD-positive patients may be a promising approach for preventing AML relapse post-transplantation, but safer clinical application principles need to be established (97).

In conclusion, the use of ICIs in the treatment of post-allo-HCT relapses holds promise, and their combination with HMAs may further enhance efficacy (98). However, it is important to note that these therapies can also carry the risk of severe side effects such as treatment-resistant GVHD and irAEs. Key risk factors for GVHD include prior history of GVHD, time since allo-HCT, ICI dosage, and transplant characteristics. Further investigation is warranted to determine the potential for improved OS or response rates. More comprehensive prospective studies are necessary to assess the safety and efficacy of ICIs in this context. Additionally, PTCy may provide a safer platform for administering ICIs after relapsed disease in both lymphomas and myeloid malignancies. Patients with a history of GVHD are at higher risk of developing GVHD after ICIs therapy. Additionally, checkpoint blocking before and shortly after transplantation increases the severity of GVHD (75, 99, 100). Considering the limited availability of alternative treatments and anticipated response rates, ICIs therapy may be considered for R/R patients, especially those without a history of GVHD who experience relapse more than 180 days after allo-HCT. Clinicians should carefully weigh the risks and benefits of using checkpoint blockade compared to other available options. Evaluating the expression of checkpoints and their ligands on immune and malignant cells is crucial for achieving positive outcomes (101). Dose optimization studies for ICIs or ICI combination therapy are currently underway in clinical trials focusing on relapse post-allo-HCT (NCT02890329, NCT03600155, NCT03912064, NCT04913922). It is recommended that the starting dose of ICIs outside of a clinical trial should be at a low dose, with close monitoring for evidence of GVHD. Immediate cessation of ICIs and rapid initiation of IV methylprednisolone at 2 mg/kg per day is recommended if GVHD occurs. If the patient does not respond rapidly to steroids, prompt initiation of second-line therapies for GVHD is advised (102). Combining two drugs may be beneficial for patients who do not respond to single ICI therapy, but it necessitates careful consideration of the potential increase in side effects (101).

## Conclusion and future directions

Although most patients with R/R hematologic malignancies responding to ICIs will experience a recurrence, allo-HCT remains a viable option for selected patients to prolong response and improve survival. Despite the risk of severe GVHD in some patients who have received ICIs prior to transplantation, studies have shown that this procedure is effective and relatively safe. The use of PTCy-based GVHD prophylaxis strategies appears to offer promising outcomes in terms of both efficacy and safety for these patients. Additionally, ICIs have shown effectiveness in the post-transplant setting; however, an increased incidence of irAEs and fatal GVHD has been reported. Clinical trials evaluating ICIs in relapse after allo-HCT are currently ongoing. The prevailing trend is to integrate ICIs with a range of pharmacological agents and therapeutic modalities. Nevertheless, the lack of comprehensive understanding of resistance mechanisms to ICIs has hindered the identification of an optimal combination therapy. Consequently, high-quality randomized controlled trial data remains scarce in this area. To address this critical knowledge gap and challenge, it is imperative to first identify safe and effective approaches for combining immunotherapies and assess their safety when used in conjunction with allo-HCT. Subsequently, prospective multicenter biomarker-related trials should be conducted.
